# The Effects of Low-Intensity Ultrasound on Fat Reduction of Rat Model

**DOI:** 10.1155/2017/4701481

**Published:** 2017-08-23

**Authors:** Bill Zhou, Benny Yuk Kin Leung, Lei Sun

**Affiliations:** ^1^David Geffen School of Medicine, University of California Los Angeles, Los Angeles, CA, USA; ^2^Interdisciplinary Division of Biomedical Engineering, The Hong Kong Polytechnic University, Hung Hom, Hong Kong

## Abstract

Nonfocused low-intensity ultrasound is generally believed to be less efficacious than High-Intensity Focused Ultrasound (HIFU) at body fat reduction; nevertheless, this technology has already been widely used clinically for body contouring purposes. This study aimed to evaluate the efficacy and safety of this new technology by applying 1 MHz nonfocused ultrasound at 3 W/cm^2^ to the outer-thigh region of rat models. Ultrasonography measurement demonstrated an average reduction of 0.5 mm of subcutaneous fat thickness that persisted for at least three days after treatment. Biochemical analysis quantified a significant increase in lipid levels, specifically triglyceride, high-density lipoprotein, and total cholesterol. These two findings of subcutaneous fat reduction and plasma lipid increase showed a positive correlation. No evidence of adverse events or complications was observed after the treatment. This study validated nonfocused low-intensity ultrasound as an effective and safe method for body fat reduction, especially with repetitive treatment. However, the concurrent increase in plasma lipid level will require further investigation to determine this technology's long-term impact, if any, on health.

## 1. Introduction

Reducing body fat is becoming an important issue in modern society due to high caloric intake and lack of exercise. Extra adipose tissue depositing around abdomen, breasts, hips, and thighs causes personal dissatisfaction with body physique and subsequent demand for effective, safe, and simple treatments for body contouring. Traditionally, liposuction was the most popular method for body contouring, but this invasive treatment has been associated with many adverse events and complications [1]. As a result, less invasive or noninvasive alternatives such as cryolipolysis [2, 3], radiofrequency ablation [4, 5], laser therapies [6], injection lipolysis [7], and ultrasound lipolysis [8, 9] have been gaining more attention due to their moderate efficacy, fewer complications, and ease of use. Among the aforementioned technologies, ultrasound lipolysis is the most novel and promising.

Ultrasound has long been utilized in medicine in two ways: diagnostic and therapeutic. One of the therapeutic applications is noninvasive adipose tissue lipolysis with either focused ultrasound or nonfocused ultrasound. High-Intensity Focused Ultrasound (HIFU) is an example of focused ultrasound that has already been shown as an effective and safe treatment in body contouring [10, 11]. With HIFU, either relatively low-intensity (17.5 W/cm^2^) or high-intensity (1000 W/cm^2^) focused ultrasound is targeted at the focal zone, causing mechanical cellular membrane disruption or coagulative necrosis of the target tissue, respectively.

In contrast, nonfocused low-intensity ultrasound at the intensity level of 0.125–7 W/cm^2^ [12] is typically used in physiotherapy for thermal treatment. It is not indicated for body contouring because it is generally believed that nonfocused low-intensity ultrasound does not have as significant or as durable of an effect when compared to the HIFU [13, 14]. In practice, however, it is widely used for noninvasive body sculpting on the assumption that its ultrasonic thermal effect may contribute to the reduction of adipose tissue while its lower intensity poses less of a health risk compared to HIFU. Despite its popularity for lipolysis, there are only a few studies on its efficacy and safety. Miwa et al. [15] used low-intensity ultrasound at 0.5 MHz with intensity level of 0.1 W/cm^2^ to treat rat abdomens for 10 minutes. After treatment, both plasma free fatty acid (FFA) and norepinephrine in the extracellular fluid around the perirenal adipose tissue were increased, indicating the ultrasound treatment can cause fat mobilization through increased norepinephrine secretion. Miwa et al. [15] also experimented with low-intensity ultrasound of 0.5 MHz and 1 MHz at 0.5 W/cm^2^ intensity on human thighs, which resulted in significantly decreased subcutaneous fat thickness but unchanged body weight. Liao et al. [16] investigated the use of 1 MHz ultrasound at 2 W/cm^2^ alone and in combination with chitosan feeding for weight reduction in mice. Test results showed that, for ultrasound treatment only, local fat pad thickness was decreased but the weight change was limited. However, with the combined treatment, both local fat pad thickness and body weight were significantly decreased. Plasma lipid levels were also tested in addition to fat pad thickness and weight change. For mice that received only the ultrasound treatment, total cholesterol level decreased while triglyceride and high-density lipoprotein levels stayed unchanged. Garcia Jr. and Schafer [17] applied 1 MHz nonfocused ultrasound at 7 W/cm^2^ and 5 W/cm^2^ on pig's abdominal adipose layer and did not find cellular debris in the lymph node tissue or the lymph node matrix after treatment. Furthermore, in the treated adipose tissue, free lipids were found in the extracellular space while the adipocytes' membranes stayed intact. These two findings suggest that low-intensity ultrasound treatment reduced the adipose tissue by changing the permeability of fat cell without causing cellular necrosis. In contrast to the two aforementioned experiments, this study demonstrated an increase in lymph fluid triglyceride, cholesterol, and high-density lipoprotein but no changes in blood lipid levels.

Through these experiments, the efficacy and mechanism of low-intensity ultrasound for body fat reduction were investigated. However, the reported changes in the plasma lipid levels in these papers were not consistent. Therefore, it is necessary to elucidate the mechanism between the decreased adipose tissue thickness and the varying blood lipid levels in order to evaluate the efficacy and safety of this new technology for body fat contouring.

In this study, 1 MHz continuous ultrasound at 3 W/cm^2^, adopted in many commercial products, was applied to the outer-thigh of 13 Sprague-Dawley rats. Ultrasonography measurement was used to quantify the subcutaneous adipose layer thickness decrease to determine the efficacy of lipolysis. Biochemical analysis was utilized to tract the changes in blood lipid levels before and after treatment. Finally, the relationship between changes in adipose tissue thickness and blood lipid levels was statically analyzed for correlation.

## 2. Materials and Methods

### 2.1. Animal Model

Three-month-old male Sprague-Dawley (SD) rats (*n* = 11, weight: 396.4 ± 28.0 g) were randomly recruited independent of body weight but excluding those with pre-existing high cholesterol disorder. During housing, the rats received a normal diet without drugs that are known to induce lipolysis in animal model. All the animal experiments in this study were conducted with ethical approval from the Department of Health, Hong Kong Special Administrative Region Government and the Hong Kong Polytechnic University.

### 2.2. Experimental Procedure

Biochemical analysis of the blood lipid level was conducted two weeks before the ultrasound treatment to allow the rats to fully recover according to the animal ethical protocol. Prior to the ultrasound treatment, general anesthesia was given to the rat subjects using 1 mL of Chloride Hydrate injected into the abdominal region with a 30-gauge needle syringe. Chloride Hydrate was chosen because it would not cause confounding effects on the measured blood parameters. Hair on the outer-thigh region was removed for better ultrasound transmission coupling. Ultrasonography measurement was conducted to record the original subcutaneous fat pad thickness in the treatment site prior to treatment.

A commercial ultrasound device (Weight Loss Model: GB-818 from Beauty® Machines) was used to treat the outer-thigh. Continuous ultrasound wave with frequency of 1 MHz and intensity of 3.2 W/cm^2^ was generated and applied to the treatment site of the anesthetized rats (*n* = 13) for 30 minutes. A layer of water-compatible conductive gel 1 to 2 cm thick was placed between the transducer and the skin to accommodate the transduction of the ultrasound wave. The ultrasound transducer was aligned by moving back and forth above the treatment site with slight compression to assist the release of fat from the adipose tissue.

**Figure 1:**
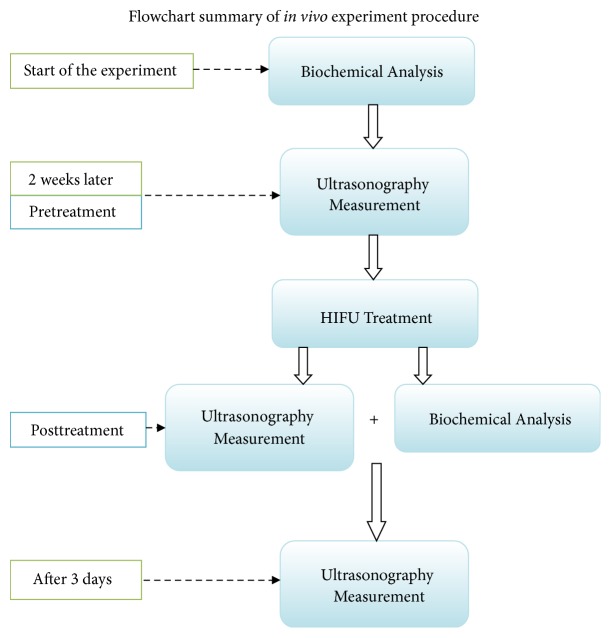
Flowchart summary of the experiment procedure.

To judge the efficacy of the low-intensity ultrasound induced lipolysis, ultrasonography measurement and blood analysis were performed again immediately after the 30-minute ultrasound treatment. To assess the durability of the lipolysis effect, ultrasonography measurement was repeated three days after the treatment as well. Flowchart of the experiment procedure is shown in Figure 1.

### 2.3. Ultrasonography Measurement

The subcutaneous fat layer thickness is defined as the vertical distance from the skin to the muscle fascia. The muscle fascia marks the boundary between the adipose tissue and underlying muscle group and is easily apparent on ultrasound as a hyperechogenic layer. A 10 MHz Sonosite 180 plus US scanner (penetration depth of 2.2 cm) was used to measure the thickness of the treatment site's subcutaneous fat pad layer before treatment, immediately after treatment, and three days after treatment. Furthermore, the thickness of the fat layer was measured with electrical caliper and averaged from three measurements at different locations. Data of the fat layer thickness from pretreatment, posttreatment, and three-day follow-up were analyzed and compared by One-Way Repeated Measure ANOVA (2-sided) using SPSS 15.0 with post hoc multiple comparison test: Bonferroni* t*-test.

### 2.4. Biochemical Analysis

Blood was collected from the anesthetized rats' lateral tail veins by venipuncture. Each lateral tail vein was pre-treated with warm water (around 35°C) to cause dilation for easier visualization followed by disinfection with 70% isopropyl alcohol. The tail vein was punctured by a 30-gauge, 8 mm needle syringe to extract 0.4 mL of blood. Squeezing was avoided to prevent the blood sample from mixing with interstitial fluid. Direct pressure was applied on the wound after venipuncture to achieve hemostasis.

Blood samples collected were transferred into 2 mL heparin tubes to prevent coagulation. Blood plasma was obtained by centrifugation (2500 rpm, 4°C, 10 minutes) within two hours to avoid degradation of blood lipids into fatty acids by red blood cell lipase activity. Blood plasma was frozen at −80°C to minimize any biochemical reaction before analysis. A biochemical analyzer (Hitachi 902) was used to measure the plasma lipid profile including triglyceride (TG), high-density lipoprotein (HDL), and total cholesterol (TC) levels.

Pre-treatment blood lipid measurements were conducted two weeks before the ultrasound treatment with the rats fasting for six hours prior to venipuncture. Posttreatment blood samples were collected from the experimental rats immediately after ultrasound treatment for analysis of whether fat was released from adipocytes. Data from the pretreatment and posttreatment blood parameters were analyzed using SPSS 15.0 with paired* t*-test (2-sided) for statistical significance.

## 3. Results

### 3.1. Ultrasonography Measurement

**Figure 2:**
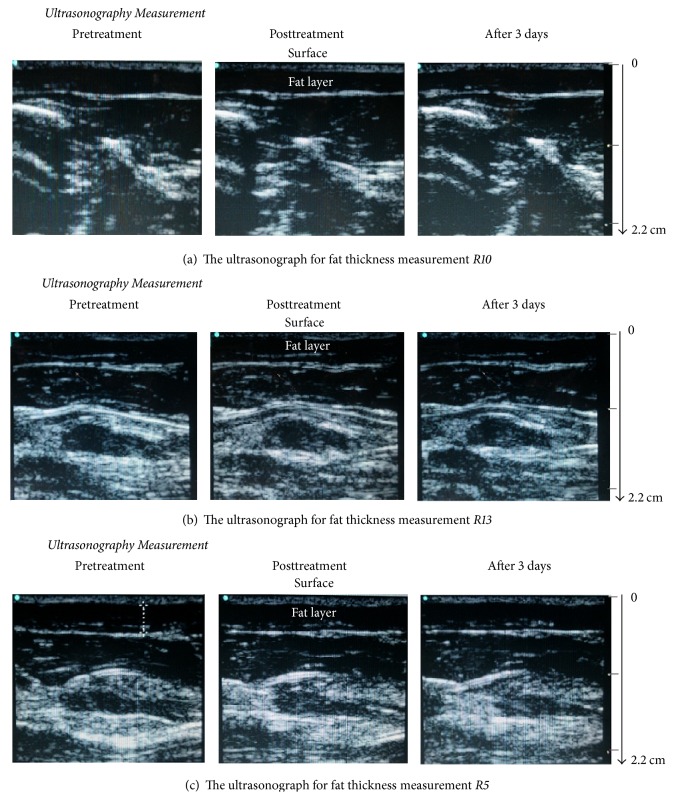
Pretreatment, posttreatment, and follow-up ultrasound images of three of the subjects (R8, R11, and R3) receiving ultrasound induced lipolysis treatment. The site of measurement was referenced to the identified anatomical trademark. (a) Fat layer showed a thickness reduction of 0.9 mm after treatment (high effect); (b) fat layer showed a thickness reduction of 0.4 mm after treatment (low effect); (c) fat layer showed a thickness reduction of 0.7 mm after treatment (Moderate effect).

Figure 2 shows the pre-treatment, posttreatment, and follow-up ultrasound images of three experimental subjects, R8, R11, and R3, which exhibited high, low, and moderate lipolysis effects from ultrasound treatment, respectively. The effect of treatment yielded inconsistent results, ranging from 0.2 to 0.9 mm reduction in the fat layer.  Table 1 summarizes the average fat layer thickness before, immediately after, and three days after ultrasound treatment. On average, the fat layer was reduced by a thickness of 0.5 ± 0.2 mm after a single 30-minute treatment with the results lasting for at least three days after treatment.

**Figure 3:**
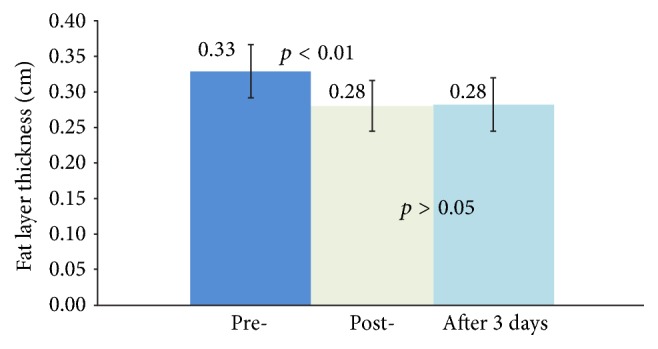
Histogram of fat layer thickness changes at different stages and the corresponding statistical significance.

Analysis for statistical significance was performed using One-Way Repeated Measures ANOVA with post hoc multiple comparison test: Bonferroni* t*-test.  Figure 3 highlights a statically significant reduction (*p* < 0.01) in the fat layer thickness between the pre-treatment and posttreatment groups, indicating that the intervention with low-intensity nonfocused ultrasound caused reduction in adipocyte thickness by inducing lipolysis. Moreover, there was not a statistically significant difference in the fat layer thickness between the immediate posttreatment and the three-day follow-up groups (*p* > 0.05), suggesting the effect of fat layer reduction persisted for at least three days.

### 3.2. Biochemical Analysis

**Figure 4:**
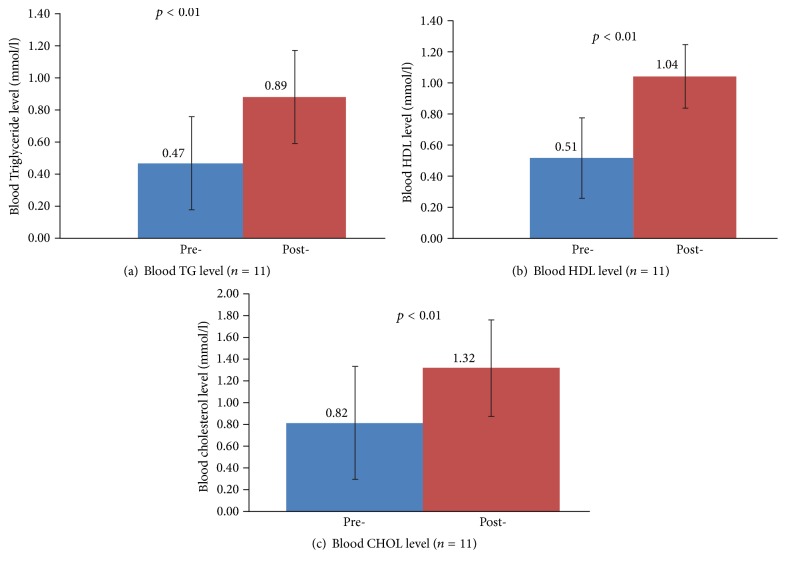
Histogram of the blood lipid level variation before and after the treatment and the corresponding statistical significance. (a) Blood TG level, (b) HDL level, and (c) CHOL level.

Table 2 shows the biochemical analysis of blood TG, HDL, and total cholesterol levels before and after treatment. On average, blood TG level increased by 0.42 ± 0.10 mmol/L, HDL increased by 0.53 ± 0.17 mmol/L, and total cholesterol increased by 0.50 ± 0.27 mmol/L after treatment. The blood lipid levels were also compared using paired* t*-test on the normally distributed data. As seen in Figure 4, there were significant increases (*p* < 0.01) in all three blood lipid levels after treatment. This suggests that the ultrasound treatment caused release of intracellular lipids into microcirculation by increasing cellular permeability of adipocytes.

### 3.3. Relationship Analysis

**Figure 5:**
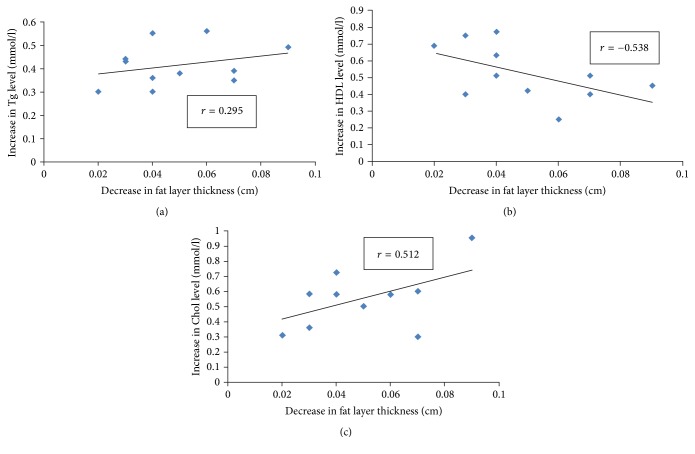
Scatter plot of the blood parameter increment and the corresponding thickness reduction. Each dot represented one of the rat subjects (*n* = 11). (a) Blood TG level, (b) HDL level, and (c) CHOL level. Correlation coefficients (*r* value) shown were 0.295 (*p* < 0.05), −0.538 (*p* < 0.05), and 0.512 (*p* < 0.05), respectively.

Statistical analysis (SPSS 15.0 Output) showed a statically significant association (*p* < 0.05) between the increased lipid levels and the reduced fat layer thickness; however, these two parameters had poor linear correlation, with a maximal correlation coefficient of less than 0.6. As seen in the correlation plots in Figure 5, the decrease in fat layer thickness is not linearly proportional to the changes in the blood lipid levels. The trend lines suggest that, in some mice, a higher reduction in fat layer thickness does not necessarily correlate with a corresponding increase in blood lipid parameters; in fact, some mice with less reduction in fat had greater increase in lipid levels. For example, R9 showed the least fat thickness reduction of all subjects but had changes in all three blood parameters comparable to that of R10, which had the largest amount fat layer reduction. On the other hand, R8, which had a moderate amount of thickness reduction, had almost the highest increase in lipid levels among all subjects.

### 3.4. Safety Examination

A thorough follow-up physical examination of the mice was benign; there were no evidences of skin burn, loss of sensation, or motor paralysis that might have been caused by the ultrasound treatment.

## 4. Discussion and Conclusions

The ultrasound settings used in this research experiment (1 MHz ultrasound at 3.2 W/cm^2^) mimicked those of therapeutic ultrasound devices claiming to be effective in body contouring. After a 30-minute treatment, the 11 rat subjects experienced an average fat layer reduction of 0.05 ± 0.02 cm (15.2% of the original thickness) in the outer-thigh region. This reduction was further maintained for at least 3 days posttreatment, indicating that low-intensity ultrasound may have a significant and durable effect on body fat reduction. However, the range of adipose tissue reduction varied from 0.2 to 0.9 mm. Different thresholds for ultrasound induced sonoporation among adipocytes may explain this lack of consistency. Individual fat cells have irregular and varying membrane compositions that result in different thresholds for increasing membrane permeability under direct ultrasound waves. Therefore, the observed variation suggests that an ultrasound intensity of 3 W/cm^2^ used in this study was inadequate to exceed the sonoporation threshold for all the adipocytes.

The biochemical analysis of plasma lipid changes provided further evidence supporting the treatment efficacy of low-intensity ultrasound in subcutaneous fat reduction. The three blood parameters, TG, HDL, and TC, all increased significantly by 89.4%, 103.9%, and 61.0% after the ultrasound treatment, respectively. These changes support the hypothesis that low-intensity ultrasound induced sonoporation in adipocytes to cause release of their lipid content into the microcirculation. Our results contrast with other studies that showed different changes in blood lipid levels after low-intensity ultrasound treatment. For example, Liao et al. [16] found that the blood TG and HDL level were relatively stable while the blood TC level decreased after treatment with 1 MHz ultrasound at 2 W/cm^2^. A possible explanation for the conflicting data is the different equipment and treatment operation used in the various experiments. In our study, 1 MHz ultrasound at 3 W/cm^2^ was applied over a 30-minute session; Liao's study used 1 MHz ultrasound at 2 W/cm^2^ in 30-second sessions over the span of five weeks. Similarly, Garcia Jr. and Schafer [17] reported there were no significant changes in blood lipid levels using 1 MHz ultrasound at 7 W/cm^2^ and 5 W/cm^2^ on pig models, which they attributed to the relatively short time between the end of treatment and the blood draw. In our study, blood was collected immediately after the ultrasound treatment in order to eliminate confounding from metabolism of free-floating lipids. We believe our observed lipid changes are caused by using a much smaller animal model (rat instead of pig) with proportionally smaller blood volume whereby a moderate release of lipids into the circulation can cause significant changes in plasma lipid levels.

As seen in Figure 5, the increase in blood lipids and decrease in fat layer thickness have a poor linear relationship, which bring into question whether there exists a correlation between the two variables. While the three blood parameters showed an overall increase, their plasma concentration did not change predictably with increased fat layer reduction as expected. However, some of the lipids had stronger correlation than others, which can be explained by the structural composition of adipocytes. Lipids generally make up 80–90% of fat cells; of these, different species of triglycerides [18] predominant in the form of lipid droplets close to the plasma membrane. Hence, triglyceride is the lipid most readily released with slight increases in membrane permeability. This volatility causes an unpredictable release of lipids into the blood with sonoporation, resulting in a nonlinear relationship with fat layer thickness reduction (*r* = 0.295,  *p* < 0.05). On the other hand, cholesterol is concentrated in the mitochondria and microsome of adipocytes. These membrane-bound organelles add an additional level of protection to the cholesterol against acoustic sonoporation. The additional membrane makes leakage of cholesterol less erratic and, in doing so, causes the lipid to be released in a more predictable manner, as illustrated by a strong linear correlation (*r* = 0.512,  *p* < 0.05). In other words, cholesterol molecules inside cellular organelles are released only if there is sufficient acoustic cavitation, so it has a stronger association with adipocyte shrinkage. As for high-density lipoprotein, there was an increase in concentration after treatment but the HDL level was actually negatively correlated to the fat thickness reduction (*r* = −0.538,  *p* < 0.05). The exact reason for the negative correlation is unclear. Recent study has reported that blood HDL level is actually mainly correlated to the physical activity of the individual [19]. Thus, different levels of physical activity across subjects may have confounded the relationship between these two variables.

Our experiment demonstrated that the 1 MHz ultrasound at 3 W/cm^2^ is effective at releasing stored lipid from targeted fat cells as well as reducing subcutaneous fat thickness. The combination of an average fat reduction of 0.5 mm from a single 30-minute session with persistent lasting effects makes low-intensity ultrasound a promising cosmetic treatment for body contouring. Furthermore, the comparatively low intensity, that is, 3 W/cm^2^, makes it much safer than the HIFU in body contouring, especially with repetitive treatments. However, prior to clinical applications, safety concerns regarding the surge in blood lipid levels must be further studied to evaluate for any long-term effects and complications.

## Figures and Tables

**Table 1:** Comparison of the fat layer thickness before and after treatment.

	Pretreatment	Posttreatment^*∗*^	After 3 days^*∗*^
Fat layer thickness/cm	0.33 ± 0.04	−0.05 ± 0.02	−0.05 ± 0.02

^*∗*^The values were represented as the thickness reduction relative to the pretreatment thickness.

**Table 2:** Comparisons of the biochemical analysis results before and after treatment.

Lipid type	Pretreatment (mmol/l)	Posttreatment (mmol/l)
Triglyceride	0.47 ± 0.28	0.89 ± 0.29
HDL	0.51 ± 0.26	1.04 ± 0.20
Cholesterol	0.82 ± 0.52	1.32 ± 0.44

## References

[1] Triana L., Triana C., Barbato C., Zambrano M. (2009). Liposuction: 25 years of experience in 26,259 patients using different devices. *Aesthetic Surgery Journal*.

[2] Avram M. M., Harry R. S. (2009). Cryolipolysis™ for subcutaneous fat layer reduction. *Lasers in Surgery and Medicine*.

[3] Klein K. B., Zelickson B., Riopelle J. G. (2009). Non-invasive cryolipolysis™ for subcutaneous fat reduction does not affect serum lipid levels or liver function tests. *Lasers in Surgery and Medicine*.

[4] Sadick N. S., Mulholland R. S. (2004). A prospective clinical study to evaluate the efficacy and safety of cellulite treatment using the combination of optical and RF energies for subcutaneous tissue heating. *Journal of Cosmetic and Laser Therapy*.

[5] Manuskiatti W., Wachirakaphan C., Lektrakul N., Varothai S. (2009). Circumference reduction and cellulite treatment with a TriPollar radiofrequency device: A pilot study. *Journal of the European Academy of Dermatology and Venereology*.

[6] Jackson R. F., Dedo D. D., Roche G. C., Turok D. I., Maloney R. J. (2009). Low-level laser therapy as a non-invasive approach for body contouring: a randomized, controlled study. *Lasers in Surgery and Medicine*.

[7] Matarasso A., Pfeifer T. M. (2009). Mesotherapy and injection lipolysis. *Clinics in Plastic Surgery*.

[8] Teitelbaum S. A., Burns J. L., Kubota J. (2007). Noninvasive body contouring by focused ultrasound: Safety and efficacy of the contour I device in a multicenter, controlled, clinical study. *Plastic and Reconstructive Surgery*.

[9] Ascher B. (2010). Safety and efficacy of ultrashape contour I treatments to improve the appearance of body contours: multiple treatments in shorter intervals. *Aesthetic Surgery Journal*.

[10] Fatemi A., Kane M. A. C. (2010). High-intensity focused ultrasound effectively reduces waist circumference by ablating adipose tissue from the abdomen and flanks: A retrospective case series. *Aesthetic Plastic Surgery*.

[11] Jewell M. L., Baxter R. A., Cox S. E. (2011). Randomized sham-controlled trial to evaluate the safety and effectiveness of a high-intensity focused ultrasound device for noninvasive body sculpting. *Plastic and Reconstructive Surgery*.

[12] ter Haar G. (1999). Therapeutic ultrasound. *European Journal of Ultrasound*.

[13] Moreno-Moraga J., Valero-Altés T., Riquelme A. M., Isarria-Marcosy M. I., de la Torre J. R. (2007). Body contouring by non-invasive transdermal focused ultrasound. *Lasers in Surgery and Medicine*.

[14] Mulholland R. S., Paul M. D., Chalfoun C. (2011). Noninvasive body contouring with radiofrequency, ultrasound, cryolipolysis, and low-level laser therapy. *Clinics in Plastic Surgery*.

[15] Miwa H., Kino M., Han L.-K. (2002). Effect of ultrasound application on fat mobilization. *Pathophysiology*.

[16] Liao A.-H., Ma W.-C., Wu M.-F. (2013). Evaluation of ultrasound combined with chitosan for the control of weight and local fat in mice. *Ultrasound in Medicine & Biology*.

[17] Garcia O., Schafer M. (2013). The effects of nonfocused external ultrasound on tissue temperature and adipocyte morphology. *Aesthetic Surgery Journal*.

[18] Weber N., Klein E., Mukherjee K. D. (2002). The composition of the major molecular species of adipose tissue triacylglycerols of rats reflects those of dietary rapeseed, olive and sunflower oils. *The Journal of Nutrition*.

[19] Skoumas J., Pitsavos C., Panagiotakos D. B. (2003). Physical activity, high density lipoprotein cholesterol and other lipids levels, in men and women from the ATTICA study. *Lipids in Health and Disease*.

